# Porcine commensal *Escherichia coli*: a reservoir for class 1 integrons associated with IS*26*

**DOI:** 10.1099/mgen.0.000143

**Published:** 2017-12-08

**Authors:** Cameron J. Reid, Ethan R. Wyrsch, Piklu Roy Chowdhury, Tiziana Zingali, Michael Liu, Aaron E. Darling, Toni A. Chapman, Steven P. Djordjevic

**Affiliations:** ^1^​ The i3 institute, University of Technology Sydney, Ultimo, NSW 2007, Australia; ^2^​ NSW Department of Primary Industries, Elizabeth MacArthur Agricultural Institute, Menangle, NSW 2568, Australia

**Keywords:** antimicrobial resistance, commensal *E. coli*, virulence, IS*26*, animal *E. coli*, microbial genomic epidemiology

## Abstract

Porcine faecal waste is a serious environmental pollutant. Carriage of antimicrobial-resistance genes (ARGs) and virulence-associated genes (VAGs), and the zoonotic potential of commensal *Escherichia coli* from swine are largely unknown. Furthermore, little is known about the role of commensal *E. coli* as contributors to the mobilization of ARGs between food animals and the environment. Here, we report whole-genome sequence analysis of 103 class 1 integron-positive *E. coli* from the faeces of healthy pigs from two commercial production facilities in New South Wales, Australia. Most strains belonged to phylogroups A and B1, and carried VAGs linked with extraintestinal infection in humans. The 103 strains belonged to 37 multilocus sequence types and clonal complex 10 featured prominently. Seventeen ARGs were detected and 97 % (100/103) of strains carried three or more ARGs. Heavy-metal-resistance genes *merA, cusA* and *terA* were also common. IS*26* was observed in 98 % (101/103) of strains and was often physically associated with structurally diverse class 1 integrons that carried unique genetic features, which may be tracked. This study provides, to our knowledge, the first detailed genomic analysis and point of reference for commensal *E. coli* of porcine origin in Australia, facilitating tracking of specific lineages and the mobile resistance genes they carry.

## Abbreviations

APEC, avian pathogenic Escherichia coli; ARG, antimicrobial-resistance gene; EHEC, enterohaemolytic Escherichia coli; EMAI, Elizabeth MacArthur Agricultural Institute; EPEC, enteropathogenic Escherichia coli; ETEC, enterotoxigenic Escherichia coli; ExPEC, extraintesintal pathogenic Escherichia coli; IPEC, intestinal pathogenic Escherichia coli; IS, insertion sequence; MDR, multidrug resistant; MLST, multilocus sequence typing; VAG, virulence-associated gene.

## Data Summary

One hundred and forty-three whole-genome sequences of porcine faecal *Escherichia coli* sequenced in this project have been deposited at the European Molecular Biology Laboratory (EMBL) European Nucleotide Archive under study accession number PRJEB21464 [https://www.ebi.ac.uk/ena/data/view/PRJEB21464]. For individual sample accession numbers, please refer to Table S1 (available in the online version of this article). Further strain data is available in Tables S2–S6.

## Impact Statement

The data presented in this manuscript describes for the first time, to our knowledge, a genomic analysis of commensal *Escherichia coli* from commercial swine-production facilities in Australia. Pig production routinely involves antibiotic use for disease treatment and prophylaxis, and feed additives containing zinc and heavy metals to control infectious disease. The study is significant because it reports phylogenetically diverse *E. coli* that are multidrug resistant (MDR; resistant to three or more classes of antimicrobials) and carry class 1 integrons altered by IS*26*. This initial descriptive work is important as a basis for the analysis of porcine faecal *E. coli* in all countries that produce swine commercially, due to the scale of global pork production and the vast quantities of faecal waste that are used as manure. The contamination of this waste with MDR bacteria, antimicrobial-resistance genes and unmetabolized antimicrobial residues is a concern. It is necessary to characterize food-chain-associated micro-organisms, such as *E. coli*, with zoonotic potential and multiple resistance genes, as they may pose a threat to public health.

## Introduction

*Escherichia coli* is the most frequently isolated Gram-negative pathogen affecting human health [1]. Isolates are frequently resistant to multiple antibiotics and modelling studies forecast that multidrug resistant (MDR; resistant to three or more classes of antimicrobials) *E. coli* infections will account for 30 % of 10 million fatal MDR infections annually by 2050 [2]. In addition to the pathogenic variants, commensal *E. coli* comprise an important component of the gut microbiota. *E. coli* are shed into the environment in high numbers. For example, each gram of faeces from commercially reared pigs contains between 10^4^ and 10^8^ *E. coli* [3]. It is important to understand the characteristics of these *E. coli* given the huge quantities of faeces generated and disseminated by intensive pig production. China, the world’s largest producer of swine, produces an estimated 0.618 billion to 1.29 billion metric tonnes of swine faeces each year [4, 5].

Pathogenic *E. coli* are broadly divided into intestinal pathogenic *E. coli* (IPEC) and extraintestinal pathogenic *E. coli* (ExPEC). ExPEC have a faecal origin, having persisted asymptomatically in the gut before opportunistically colonizing extraintestinal sites where they cause a diverse range of diseases, including urinary-tract infections (UTI), pyelonephritis, wound infections, sepsis and meningitis [6]. ExPEC are thought to have foodborne reservoirs and may enter the food chain via a number of sources [7–11]. The zoonotic potential of commensal porcine *E. coli* as a source of ExPEC that cause disease in humans is unknown. ExPEC cannot be reliably detected in a diagnostic test as they are yet to be shown to possess unique identifying features relative to other pathotypes of *E. coli* [12]. Instead, as we aim to do here, whole-genome sequencing can be used to discriminate strains indistinguishable by other methods, and identify any genetic relationships between *E. coli* strains isolated from pigs and humans.

Horizontal gene transfer, mediated by mobile genetic elements, plays an important role in the evolution of *E. coli*. Commensal or pathogenic bacteria may, in a single horizontal gene transfer event, acquire a mobile genetic element carrying multiple antimicrobial-resistance genes (ARGs), virulence-associated genes (VAGs) and other genetic cargo that encode traits that offer a niche advantage [13–17]. The release of MDR commensal *E. coli* into the environment, such as when pig faeces are used as manure, facilitates horizontal transfer of resistance and virulence genes into other microbial communities in a manner that is poorly understood. ARGs cluster on mobile genetic elements and form complex resistance regions that are often independently mobile. Indirect selection pressure can, in the absence of antibiotic use, lead to the persistence of transferred genes. For example, heavy metals such as copper and zinc in feed formulations for food animals select for ARGs that co-localize with metal-resistance genes [18, 19]. Selection pressure afforded by any one of a number of antibiotics and heavy metals (zinc, cadmium, mercury) that contaminate faecal waste or those used in food-producing and hospital environments is sufficient to select for the retention and spread of complex resistance regions [20, 21]. Understanding of how ARGs assemble on mobile genetic elements, and the extent to which these then traffic through human, food animal and environmental reservoirs, remains limited.

Class 1 integrons are a reliable proxy for the presence of multiple ARGs within bacteria in clinical and veterinary settings [22]. They are gene capture and expression elements that can integrate AMR gene cassettes from the environmental resistome and express them via a promoter residing in the class 1 integrase gene. They are often mobilized by mercury-resistance transposons belonging to the Tn*21* family, which have been disseminated globally on a wide variety of conjugative plasmid backbones [23]. Resistance genes can also be acquired, lost and rearranged in bacteria by genetic events that involve insertion sequences (ISs) such as IS*26*, IS*Ecp1*and IS*CR1* [24–27]. IS*26* is prominent in this regard due to its unique mechanisms of transposition (conservative and replicative), ability to recognize itself, lack of copy number control and ability to mobilize a wide range of ARGs [15, 26, 28–30]. Furthermore, IS*26* is recognized to play a key role in: (i) the evolution of plasmids and genomic islands that carry combinations of VAGs and ARGs [16, 17, 31, 32]; (ii) driving the formation of cointegrate plasmids encoding VAGs and ARGs [33]; and (iii) initiating deletions in large multidrug-resistance plasmids that enhance plasmid stability and expand host range [34].

Infectious-disease management relies on the surveillance of antimicrobial resistance and emerging pathogens using a One Health approach. There is currently no published data available that records whole-genome-sequence-based phylogeny, or ARG or VAG carriage in commensal *E. coli* from Australian pigs, and only one comparable study is available from overseas [35]. Here, for the first time, to our knowledge, we present whole-genome sequence analysis of 103 class 1 integron-positive commensal *E. coli* from pigs commercially reared in Australia. We present data characterizing their phylogenetic diversity, carriage of VAGs and ARGs, and an analysis of the class 1 integrons they carry.

## Methods

### Management of farms and animals

The study was conducted using *E. coli* sourced separately from two pig-production farm systems located approximately 250 km apart. Farms were designated descriptors F1 and F2. Isolate numbers consist of farm number, a pig number and a letter designating a single isolate from that pig (i.e. F1_404D indicates farm 1, isolate D from pig 404). At both farms, pigs were intensively housed and kept in total confinement. Both farms have used neomycin in the past for the treatment of diarrhoeal disease. No antibiotics were being used during the first sampling time at F1; however, the pigs sampled at the second sampling time had received a course of neomycin (see below). No antibiotics had been administered to the pigs at F2 prior to sampling.

### *E. coli* strains used in the study

*E. coli* isolates were collected via rectal-swab sampling of pigs between 19 and 30 days of age. At farm 1, rectal swabs were collected in May 2007 from pigs during an outbreak of diarrhoeal disease, but prior to treatment with neomycin. These pigs were subsequently removed from the shed. The causative agent of the outbreak was unknown. A new batch of healthy sows and their piglets were transferred to this shed and the sows were given neomycin in-feed. Once the piglets were weaned they also received neomycin in-feed for 7–10 days. The second sampling occurred on these piglets in June 2007 after the course of antibiotics. At farm 2, rectal swabs were performed on healthy weaners that were not treated with antibiotics.

*E. coli* were isolated at the Elizabeth MacArthur Agricultural Institute (EMAI), Australia. Up to ten *E. coli* colonies were selected from individual pigs using MacConkey agar. The total collection from farm 1 was 164 isolates from 33 pigs, whilst from farm 2 was 171 isolates from 23 pigs. All strains were screened by PCR for the class 1 integrase gene *intI1*. This screening indicated that 117/164 (71 %) *E. coli* from farm 1 and 168/171 (98 %) from farm 2 carried *intI1.* Initially, 50 *intI1*-positive isolates from F1 and 100 *intI1*-positive isolates from F2 were selected for whole-genome sequencing. Two enterotoxigenic *E. coli* (ETEC) strains, M10 and ETEC286_3, which were submitted to the EMAI from Australian veterinary services, as clinical, pig-derived strains, were also sequenced and included in the phylogenetic analysis as reference strains.

### Storage

All strains were freshly cultured in LB medium and frozen as glycerol stocks made using 500 µl M9 salts solution and 500 µl 50 % (v/v) glycerol and stored at −80 °C. All strains were cultured in LB medium prior to isolation of total cellular DNA used for sequencing.

### DNA extraction, whole-genome sequencing and assembly

Total DNA was extracted using the ISOLATE II genomic DNA kit (Bioline) following the manufacturers standard protocol for bacterial cells and stored at −20 °C. Whole-genome sequencing libraries were prepared from separate aliquots of sample DNA using the Illumina Nextera DNA kit with modifications. In brief, the DNA was first quantified using a Qubit dsDNA HS assay kit (Thermo Fisher Scientific). All sample DNA concentrations were standardized to equal concentration to achieve uniform reaction efficiency in the tagmentation step. Standard Illumina Nextera adaptors were used for sample tagmentation. The PCR-mediated adapter addition and library amplification was carried out using customized indexed i5 and i7 adaptor primers (IDT), which were developed based on the standard Nextera XT indexed i5 and i7 adapters (e.g. N701–N729 and S502–S522). Libraries were then pooled and size selected using SPRI-Select magnetic beads (Beckman Coulter). Finally, the pooled library was quality checked and quantified on an Agilent Bioanalyzer 2100 using the DNA HS kit (Agilent). Whole-genome sequencing for the majority of F1 strains and ETEC strains was performed as previously reported [36], using an Illumina MiSeq sequencer and MiSeq V3 chemistry. Whole-genome sequencing of the remaining F1 and F2 strains was performed using an Illumina HiSeq 2500 v4 sequencer in rapid PE150 mode. Sequence read quality was initially assessed using FastQC version 0.11.5 (http://www.bioinformatics.babraham.ac.uk/projects/fastqc/). Illumina raw reads passing quality control were assembled into draft genome sequences using the A5 assembly pipeline, version A5-miseq 20140604 [37]. Genome sequences have been deposited in the European Molecular Biology Laboratory European Nucleotide Archive with study accession number PRJEB21464. Accession numbers for each sample are listed in Table S1.

### Strain selection

Sequence data was successfully generated for 141 strains and these were screened by blast for *intI1,* ARGs and VAGs, and subjected to Phylosift analysis as described below. These analyses indicated 12 strains were negative for *intI1* and that a number of clones were isolated from individual pigs. We, therefore, excluded *intI1-*negative strains and selected representatives of the clonal isolates, thereby excluding a further 26 strains. The subset of strains that were sequenced were identified as F1+F2 (*n*=103 from 42 pigs). This subset consisted of 35 strains from 21 pigs sampled at farm 1 and 68 strains from 21 pigs sampled at farm 2; among the F1 strains, 17 were disease-associated strains from 12 pigs (isolate numbers 1–30, designated ‘disease’ in Tables S1–S5) and 18 were isolated from 11 healthy pigs (isolate numbers 365–409, designated ‘healthy’ in Tables S1–S5). Only 11 isolates in the collection carried toxin genes (*eltA*, *n*=2; *eltB*, *n*=2; *stA*, *n*=0; *stB*, *n*=11) associated with porcine ETEC and no ETEC adhesins were detected. Notably, only five of these were from diseased pigs, whilst six were from healthy pigs. This highlights the role that host factors, such as stress and immune health, play in the manifestation of pre- and post-weaning diarrhoea in pigs and we, therefore, argue that this collection should be considered commensal.

### Assembly statistics

Comprehensive assembly statistics for 143 sequenced porcine-derived *E. coli,* (141+2 ETEC) are available in Table S1. Isolates not included in this study are highlighted grey. The number of scaffolds per genome ranged from 29 to 1571, with a mean of 235. Each genome sequence had a median sequencing coverage of at least 20 ×, with a maximum of 94× and mean of 54×.

### Phenotypic resistance testing

F1 strains were tested at the EMAI using the calibrated dichotomous susceptibility (CDS) test for resistance to 12 antibiotics [38]. The following were tested: ampicillin (25 µg), cefoxitin (30 µg), nalidixic acid (30 µg), ciprofloxacin (2.5 µg), imipenem (10 µg), sulphafurazole (300 µg), trimethoprim (5 µg), tetracycline (10 µg), neomycin (30 µg), gentamicin (10 µg), azithromycin (15 µg) and chloramphenicol (30 µg). F2 strains were tested for resistance to antibiotics at the i3 Institute, University of Technology Sydney, Australia, using the same method and panel of antibiotics as the F1 collection. F2 strains were also tested with streptomycin (25 µg) and kanamycin (50 µg) (Table S2).

### Gene identification and serotyping

Resistance, virulence and plasmid-associated genes were identified using local blastn v2.2.30+ [39] searches with an *E* value of 1.0×10^−3^ (Tables S3–S5). The gene databases used were ResFinder, PlasmidFinder, ISFinder, SerotypeFinder and VirulenceFinder [Data references 1–5] [40–44]. Our virulence database was supplemented with additional virulence genes from GenBank, available in Table S6. Genes were considered present if the subject nucleotide sequence was >90 % identical over 100 % of the length of the query sequence. blast hits with >90 % identity but covering less than 100 % of the query were considered positive if they were truncated by a scaffold break or insertion. Integrons were characterized in SnapGene (GSL Biotech) using blastn output. The collection was then retroactively screened for characterized integrons using blastn. Where strains carry two *intI1* genes, *de novo* assembly software is unable to assemble the two complete integrons with Illumina short read data, as it cannot determine which cassette array belongs to which *intI1* copy. The presence of two integrons in strains in this collection was, therefore, initially inferred by blast identification of their cassette arrays and downstream regions (e.g. IS*26* deletion signatures), and then confirmed by read-mapping using Bowtie2 and Tablet [45, 46].

### Phylogrouping and multilocus sequence typing (MLST)

*E. coli* phylogroups were determined using the scheme published by Clermont *et al.* [47]. The genes *chuA* (GenBank accession no. U67920.1)*, yjaA* (GenBank accession no. NC_000913.3) and the DNA fragment TspE4.C2 (GenBank accession no. AF222188.1) were sourced from GenBank and identified *in silico* using blastn. MLST was performed *in silico* using the PubMLST database (http://pubmlst.org/) and the Achtman *E. coli* MLST scheme (http://mlst.warwick.ac.uk/mlst/).

### Phylogenetic analyses

Maximum-likelihood phylogenetic distances between genomes were analysed using the PhyloSift pipeline [48], and a tree was generated using FastTree2 [49]. The tree was visualized using FigTree v1.4.2 (http://tree.bio.ed.ac.uk/software/figtree/) and iTOL (https://itol.embl.de/). The FastTree2 protocol was modified to resolve short branches, as described previously [50].

## Results

Our study collection consisted of 103/335 (31 %) strains of *E. coli* isolated from rectal swabs of pigs from two farms in New South Wales, Australia, that were PCR-positive for the class 1 integron integrase gene, *intI1.* Initial screening indicated that 117/164 (71 %) *E. coli* from farm 1 and 168/171 (98 %) from farm 2 carried *intI1*.

### Population structure of *E. coli* isolated from porcine rectal swabs

Strains in our study collection were classified by phylogrouping, *in silico* MLST and *in silico* serotyping. The majority of the strains in our study collection 74/103 (72 %) belonged to phylogroup A, while the remainder belonged to phylogroup B1 (18; 17 %), phylogroup B2 (5; 5 %) and phylogroup D (6; 6 %).

**Fig. 1.**
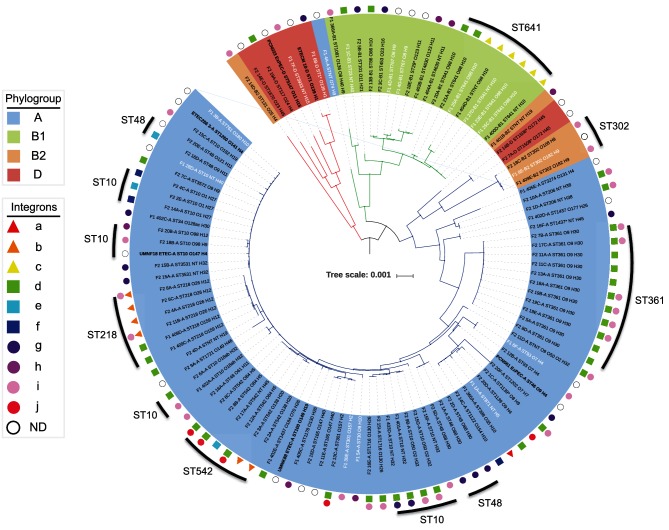
A mid-point rooted, maximum-likelihood phylogenetic tree inferred using PhyloSift v1.0.1, FastTree2, FigTree v1.4.2 and iTOL. The tree contains all 103 pig *E. coli* isolates sequenced in this study, 2 porcine ETEC strains and 4 reference pig-sourced sequences. The labels of strains isolated from pigs with diarrhoea are in white, and of ETEC and reference strains are in bold. Branches are coloured by clade (clade 1, red; clade 2, green; clade 3, blue). Shading over tip labels indicates phylogroup (A, blue; B1, green; B2, orange; D, red). Tip labels also contain multilocus sequence type and serotype. Asterisks indicate single-locus variants of a given sequence type. The tree scale shows the distance for 1 amino acid substitution per 1000 sites in the analysis. Clusters of the seven most common sequence types have been marked with an outer line. Integrons shown in Fig. 3 are annotated by shapes indicating the presence of *sul1* (triangles), IS*26-*truncated 3′-*CS* (squares) and *sul3* (circles). Integrons (a–j) are coloured red, orange, yellow, green, aqua, blue, purple, magenta, pink and crimson. Strains that were *intI1* positive, but were not characterized are annotated with a white circle. Integrons were not determined for reference genomes used in the analysis.

We identified 37 distinct sequence types, 21 of which were previously isolated from swine, as reported by the *E. coli* MLST database (http://mlst.warwick.ac.uk/mlst/dbs/Ecoli; accessed June 2017). Only seven sequence types were common to both F1 and F2. The most prominent sequence types were ST10, ST361, ST641, ST542, ST48 and ST218. Twenty-five sequence types were represented by a single isolate. Six strains with a single SNP in a reference allele were assigned putative sequence types (Fig. 1, Table S3; denoted by an asterisk). A designation of non-typable was given to the five remaining strains for which one or more alleles could not be determined.

*In silico* O:H typing using SerotypeFinder predicted 47 serotypes for 85 strains. The remaining 18 strains were O-non-typable with 10 different H types (Fig. 1, Table S3). In general, strains of any given sequence type carried the same O:H alleles, though intra-sequence type variability was observed among eight sequence types (ST10, ST48, ST218, ST542, ST641, ST302, ST4630 and ST1437).

### Phylogenetic analysis

To determine genetic relatedness, we used PhyloSift, FastTree2, FigTree v1.4.2, and iTOL to generate and visualize a mid-point rooted, maximum-likelihood phylogenetic tree containing the F1+F2 pig *E. coli* draft whole-genome sequences, two ETEC strains (ETEC286_3 and ETECM_10) and four pig-pathogenic *E. coli* complete genome sequences [*E. coli* UMNK88 (NC_017641.1), UMNF18 (NZ_AGT D01000001.1), PCN033 (NZ_CP006632.1) and PCN061 (NZ_CP006636.1)] (Fig. 1). Tree topology was highly congruent with Achtman MLST and *in silico* serotyping, grouping strains by sequence type, and then further by serotype. Clade structure was generally congruent with phylogroup analyses; however, seven strains belonging to phylogroups B2 and D formed a separate clade. We identified three major clades, with the seven B2/D phylogroup strains forming clade 1. Clade 2 consisted almost exclusively of phylogroup B1 strains, ST641 was the dominant sequence type; however, one phylogroup A strain (F1_4A) was an unexpected member of this clade. Clade 3 was composed of two separate sub-clades, one consisted of six B2 and D strains (three ST302, two ST1508 and a non-typable) and the other exclusively containing phylogroup A strains (ST10 and sequence types within CC10, as well as ST361 and ST542, strains that were common in our study collection).

### ARGs and heavy-metal-resistance genes

**Fig. 2.**
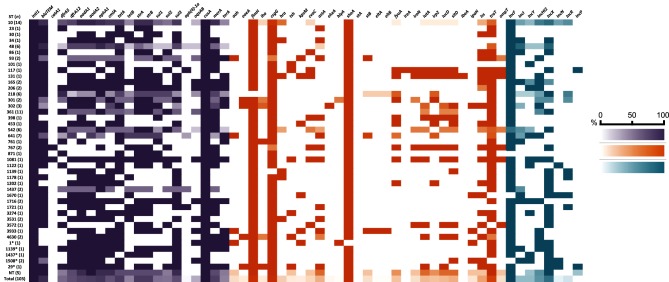
Heat map depicting carriage of ARGs (aqua), VAGs (orange) and plasmid incompatibility groups (purple) by sequence type. A darker colour indicates high carriage amongst a given sequence type, a lighter colour indicates lower carriage and white indicates no carriage. For full screening data see Tables S3–S5.

We identified a total of 17 ARGs in the collection and strains carried between 1 and 15 ARGs each. A total of 100/103 (97 %) strains carried three or more resistance genes. Surprisingly, strains belonging to phylogroup A carried the highest mean number of ARGs (10 per strain). Strains belonging to phylogroup B1, B2 and D each carried a mean of eight ARGs per strain. The most common ARGs among the strains in our collection were: the penicillin-resistance gene *bla*_TEM-1_, (84; 82 %); *aphA1,* encoding resistance to kanamycin and neomycin (76; 74 %); the co-linked streptomycin-resistance genes, *strA* and *strB* (73; 71 %); and the tetracycline-resistance gene *tetA* (73; 71 %). Quinolone-resistance genes *oqxAB,* which typically localize on plasmids, were less frequently identified (27; 26 %). Genes encoding extended-spectrum β-lactamases, extended-spectrum carbapenemases and resistance to macrolides were not detected. Heavy-metal-resistance genes, including the copper-resistance gene *cusA* (103; 100 %), the Tn*21* mercury-resistance gene *merA* (71; 69 %) and the tellurite-resistance gene *terA* (40; 39 %), were identified frequently (Fig. 2).

**Fig. 3.**
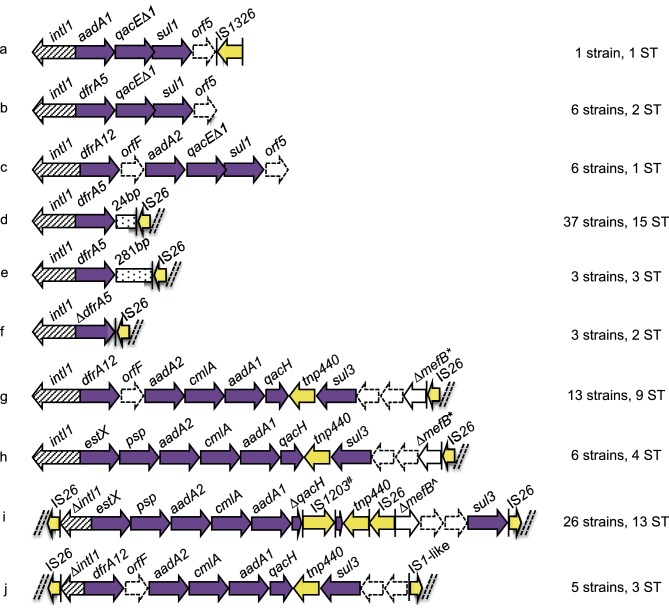
Schematic diagram (not to scale) of integrons within porcine strains that were sequenced. Arrows represent ORFs. Arrows with broken lines indicate hypothetical proteins. Vertical bars represent inverted repeats. Dashed double diagonal lines represent sequence scaffold breaks. Intergenic sequences are not shown. ARGs (purple) and IS/transposable elements (yellow) are colour coded. *, 260 bp of *mefB* remaining; ^, 111 bp of *mefB* remaining; ^#^, IS*1203-*like.

Five ARGs were identified as gene cassettes carried by class 1 integrons (Fig. 3). Cassettes carried by the majority of strains included those conferring: aminoglycoside resistance, *aadA1* (69; 67 %) and *aadA2* (72; 70 %); chloramphenicol resistance, *cmlA* (60; 58 %); and trimethoprim resistance, *dfrA12* (62; 60 %) and *dfrA5* (51; 50 %). Among sulphonamide-resistance genes, *sul3* was identified in more strains (62; 60 %) than *sul1* (48; 47 %) or *sul2* (46; 45 %) (Fig. 2, Table S3). *sul1* and *sul3* were associated with integrons (Fig. 3).

### Structurally diverse class 1 integrons

Among our study collection, we sought to characterize the diversity of class 1 integrons present. It is challenging to assemble complete sequences for such regions using Illumina sequence data, because of the presence of repeated elements. However, we identified numerous structurally diverse class 1 integrons, hereafter referred to as integrons (a–j) (Fig. 1 and 3). Notably IS*26* altered the 3′ region in six of the most common structures (d–i).

Four different class 1 integrons (g–j) carried a *sul3* gene. The first time *sul3* was linked with *E. coli* from a food-animal source in Australia was in 2015 in a highly virulent porcine ST4245 ExPEC strain [50]. Moreover, *sul3* was first reported in a human in Australia in 2017 in a commensal *E. coli* ST95 [51]. In integrons (g) and (h), the *sul3* module, which comprises a putative transposase *tnp440, sul3,* two hypothetical proteins (*orfA* and *orfB*) and 260 bp of the macrolide efflux gene *mefB* truncated by IS*26*, was the same. Integrons (g) and (h) differed from each other in their respective cassette arrays. Integron (i) differed from (g) and (h) both in its *sul3* module, which carried an additional copy of IS*26,* length of the *mefB* gene fragment (111 bp) and an insertion of an IS*1203-*like element in *qacH*. In integron (j), an IS*26* insertion leaves only 197 bp of *intI1* remaining, *mefB* is absent and an IS1-like element is adjacent to *orfB.* Only three of the integrons (a–c) among our strain collection carried a *sul1* gene. Screening indicated that at least 22 strains carry two integron structures. The most common co-carriage pattern was (d, i) (14/22), though (b, i) (2/22), (d, j) (4/22) and (d, g) (2/22) also occurred (Fig. 1, Table S3). Eight sequence types carried more than one integron, including predominant types ST10, ST361 and ST542 (Table S3).

### VAGs

To assess the virulence potential of commensal pig *E. coli* strains in our collection, we screened for a total of 94 genes that have been associated with either intestinal disease or extraintestinal disease caused by *E. coli* pathotypes. Twenty-nine of these genes were present in at least one strain (Fig. 2, Table S4). All strains possessed between 3 and 16 VAGs. The mean number of VAGs for each phylogroup was: A, 5; B1, 9; B2, 11; D, 9. The VAGs were present in diverse gene combinations between and within sequence types. Most VAGs were typical of extraintestinal *E. coli* pathotypes (ExPEC), whilst ETEC toxin gene (*eltA, eltB, stA, stB*) carriage was only observed in 11 strains and no ETEC adhesins were present.

### Plasmid incompatibility groups

We screened the collection for plasmid replication-associated genes from nine plasmid incompatibility groups that are commonly associated with carriage and mobility of ARGs. IncF was the most common replicon (89; 86 %), followed by IncX (61; 59 %) and IncHI2 (43; 42 %). All replicons were present across multiple sequence types (Fig. 2, Table S5).

## Discussion

Globally, there is a poor representation of genomic sequences for commensal *E. coli* isolated from the faeces of pigs, and none in Australia. Here, for the first time, to our knowledge, we have sequenced the genomes of *E. coli* isolated from the faeces of predominantly healthy pigs and determined their Clermont phylogroup, multilocus sequence type (Achtman) and serotype, as well as carriage of ARGs and VAGs. The phylogenetic relationships shared by the 103 strains, the types of resistance genes that reside within the class 1 integrons and the structures of class 1 integrons were also investigated. Despite sampling only two commercial piggeries, we identified a wide variety of multilocus sequence types. The diversity of isolates differed to previous studies on *E. coli* in pigs [35, 52] and this may be due to our selection of *intI1-*positive strains or simply reflect geographical differences. Our findings suggest that commensal *E. coli* populations residing within the faeces of pigs are often resistant to multiple antimicrobial agents and carry numerous VAGs. Notably, we also identified genetic epidemiological markers for tracking antimicrobial-resistance loci residing on mobile genetic elements in commensal *E. coli*.

### Commensal *E. coli* lineages are associated with disease

The dominant lineages in our collection were phylogroup A *E. coli* belonging to sequence types residing within CC10, particularly ST10, ST48 and ST218. ST10 has previously been reported as the dominant sequence type from pigs in Germany, Denmark, Ireland and Spain [3, 35, 52–54]. Our data and the observations of others suggest *E. coli* of CC10 sequence type may be opportunistic, MDR pathogens with a broad animal host range. *E. coli* CC10 can colonize humans, swine, poultry, dogs, migratory birds, rodents, camels and cattle [9, 12, 55–61]. *E. coli* CC10 can also be isolated from raw and treated wastewater, and from urban streams [8]. *E. coli* CC10 is increasingly associated with intestinal disease in humans [62, 63], and extraintestinal infections in pigs [64, 65], dogs [57] and humans, including UTI, pyelonephritis and sepsis [9, 66–68]. *E. coli* CC10 are often MDR, and the resistance genes they carry can encode resistance to extended-spectrum β-lactams [69, 70]. ST10 is a noted ExPEC sequence type in humans and has been identified in food animals, retail meats and the environment [58, 71–74]. The core attributes of ST10 that enable it to colonize diverse niches remain unknown. The phylogenetic diversity we observed within porcine faecal ST10 suggests that such attributes may vary between strains. Whole-genome sequence analysis of *E. coli* ST10 genomes from different regions of the world and from different hosts is needed to understand the full diversity and success of this sequence type.

### MDR porcine *E. coli* carry structurally diverse class 1 integrons

Notably, *sul3* was the most frequently identified *sul* gene in our collection and three different *sul3*-containing integron structures were identified. Carriage of class 1 integrons possessing *sul3* has been observed in disease-associated and commensal *E. coli* isolates from animals and humans, as well as in bacterial species other than *E. coli* from different countries [75–77]. In Australia, the carriage of *sul3* by *E. coli* has been reported infrequently, although it has been identified in several uropathogenic *E. coli* isolates [78], in a highly virulent porcine ST4245 ExPEC strain [50], and in a human commensal ST95 *E. coli* on a virulence plasmid that carries multiple ARGs and VAGs [14]. In Europe, class 1 integrons containing *sul3* have been observed in commensal *E. coli* from both humans and animals, indicating they are widely disseminated in a variety of *E. coli* lineages [14, 79–82]. Structures similar to ours have also been reported in different *Salmonella enterica* serovars, suggesting inter-species transfer of class 1 integrons carrying *sul3* may have occurred [75].

**Fig. 4.**
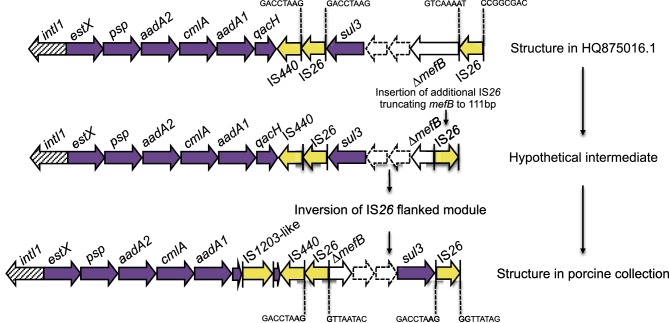
Schematic diagram (not to scale) of proposed evolutionary pathway to the *sul3-∆mefB* arrangement shown in Fig. 3(i). IS*26* 8 bp direct repeats are annotated.

The potential role for *sul3* integrons in intraspecies and interspecies exchange of antibiotic resistance makes it desirable both to understand their evolution and to track their movement through bacterial populations. In Fig. 4, we have provided a model that could explain the micro-evolutionary events that created the novel *sul3* integron depicted in structure (i). This integron likely evolved from a progenitor similar to one described by Curiao *et al.* in a human-derived extended-spectrum β-lactamase positive *E. coli* on an IncI1 plasmid from Spain (GenBank accession no. HQ875016.1), as this is the only report to describe IS*26* adjacent to *sul3* [76]. Conceivably, the novel structure (i) emerged from insertion of a second copy of IS*26,* which further truncated *mefB,* followed by an inversion event. To our knowledge, this is the first study to identify a 111 bp *mefB* variant. Integron (i) was observed within the collection in 26 *E. coli* strains of different sequence types, suggesting horizontal transfer of a mobile element(s) carrying the integron, though we were unable to determine which mobile elements were responsible for this. Further work is needed to examine this hypothesis.

IS*26*-mediated deletions of *mefB* can be used to track *sul3*-containing integrons and additional resistance genes they may acquire due to the unique ability of IS*26* to target itself [26]. A number of different truncated variants of the *mefB* gene are carried by *sul3* integrons found in human- and animal-derived *E. coli* [14, 75–77]. Our data suggests the class 1 integrase upstream of the *sul3* module is likely to be functional based on the presence of different antibiotic cassette arrays associated with a 260 bp *mefB* deletion (g, h). blastn analysis identified *sul3* integrons carrying ∆*mefB* with an identical 260 bp deletion in porcine isolates P328.10.99.C2 (GenBank accession no. FJ196386.1) and P528.10.99.C4 (GenBank accession no. FJ196388.1) from Great Britain, though the associated cassette arrays were not completely characterized [77]. Furthermore, plasmid pCAZ590 (GenBank accession no. LT669764.1) isolated from poultry in Germany carried an identical integron (*estX-psp-aadA2-cmlA-aadA1-qacI-tnp440-sul3-orf1-orf2-∆mefB:*260bp-IS*26*) to 4(h) with an additional *bla*_SHV-12_ gene 73 bp upstream of IS*26* [83]. Although the evolutionary events that lead to this derivative structure are not known, this plasmid illustrates how IS*26* augmented integrons continue to evolve and acquire genes that confer resistance to critically important human antibiotics.

The deletion event in the 3′-*CS* of the integron depicted in (d) (*dfrA5-*IS*26*) may serve as another genetic signature for tracking resistance genes, and bacteria that carry them, through different hosts and environments [15, 84–86]. Previously, we observed the integron structure (d) on plasmids carrying VAGs in atypical EPEC strains isolated from cattle with gastrointestinal disease and *E. coli* strains linked to EHEC O26:H− isolated from a human patient with haemorrhagic colitis [16, 17]. In each of these earlier cases, the IS*26* that interrupted the 3′-*CS* of the integron formed part of the left boundary of Tn*6026,* an IS*26*-flanked, globally disseminated transposon that harbours multiple ARGs [15–17, 87, 88]. Twenty-seven strains carrying integron (d) possess the resistance genes present in Tn*6026* (*bla*_TEM_, *sul2, strAB, aphA1*) suggesting this transposon is also carried in our collection, though further studies are necessary to confirm this. This again highlights that tracking IS*26* deletions is useful for tracking not only the integrons they interrupt, but also additional resistance genes that may be acquired in association with the IS*26.*

The carriage of more than one integron in a number of prominent sequence types in the collection suggests that plasmid or transposon-mediated horizontal transfer of resistance determinants may occur within the microbiota of the porcine gut. This transfer is likely mediated by plasmids present in the collection, though transposons and IS elements may be involved. Long-read sequencing is required to test this hypothesis.

### Zoonotic potential of commensal. *E. coli* from swine

In considering the zoonotic potential of pig faecal *E. coli*, we determined the proportion of strains in our collection that carried IPEC and ExPEC VAGs. A limitation of investigating zoonotic potential for extraintestinal disease is the genetic redundancy identified in the virulence attributes from ExPEC. A recent study suggested that the number of virulence factors carried by an ExPEC strain is the only independent factor that can explain extraintestinal virulence in a mouse model of sepsis [89]. Our collection contained two strains possessing large numbers of VAGs, belonging to ST131 and ST117, representative of pandemic ExPEC clones that cause hospital- and community-acquired infections in humans worldwide [58, 90, 91]. They have both been linked with poultry and have only rarely been isolated from porcine sources [9, 58]. The single ST131 strain in our porcine collection carried 10 ARGs and 16 VAGs. The ST117 strain carried 8 ARGs and 16 VAGs, including the full array of iron-acquisition genes *fyuA, irp2, ireA, iroN, iutA, iucD* and *sitD.* Several of these genes are typically encoded on virulence plasmids circulating in APEC [92] and this profile is similar to ST117-O111:H4 strains from poultry reported by Mora *et al.* [93]. The presence of ST117 and ST131 in our collection is intriguing, and warrants further investigation.

Most of the VAGs identified in our collection were those associated with the ability to cause extraintestinal disease in humans, as well as intestinal persistence [6, 94]. Carriage of genes that are under positive selection in uropathogenic *E. coli* [95], such as heat-stable agglutinin gene *hra* [96], murine uroepithelial cell adhesin gene *iha* [97], iron-acquisition genes *fyuA, iutA, iucD* and *sitD*, and the serum survival genes *iss* and *traT*, suggest that some strains may be capable of causing extraintestinal disease in humans. Conversely, it also highlights how many ExPEC VAGs can be considered important intestinal fitness factors. The most intriguing IPEC VAG was intimin gene *eaeA*, found in eight strains, that is characteristic of several intestinal *E. coli* pathotypes, including EHEC, EPEC and atypical EPEC [98]. These strains also carried ExPEC VAGs and may represent hybrid pathotypes.

The frequency of VAGs in phylogroups A and B1, a mean of 5 and 9 VAGs per isolate, respectively, was unexpected because *E. coli* belonging to phylogroups A and B1 are considered to have low virulence potential [99, 100]. The carriage of multiple VAGs in pig *E. coli* is consistent with earlier studies [101, 102]. In China, ExPEC have been isolated from a variety of tissues and bodily fluids of pigs with septicaemia, meningitis and respiratory disease with increasing frequency since 2004 [64, 65]. It is notable that 35 % of 81 isolates in one of these studies belonged to phylogroup A, clonal complex 10 [64]. In European wild boars, which are assumed to be ancestors of domestic pigs in Europe [103], *E. coli* strains carry, on average, 7 or more VAGs, with some strains carrying up to 16 VAGs [104]. Collectively, these observations suggest that *E. coli* phylogroup A and B1, at least those sourced from swine, carry multiple VAGs.

### Contribution of food-production animals to the evolution of pathogens and antimicrobial resistance

MDR *E. coli* carrying ARGs associated with mobile genetic elements and VAGs are released into the environment by food-production animals via faecal effluent. In Australia, the capacity for pig production to contribute to the evolution and dissemination of pathogens and ARGs is restricted compared to that of pig-production systems in many other countries, due to a range of factors. Firstly, Australia has a large landmass that is surrounded by ocean, preventing the movement of animals from neighbouring countries. Secondly, importation of food animals into Australia has been restricted since the 1970s [105]. Thirdly, antibiotics such as fluoroquinolones cannot legally be administered to food animals and many others are restricted from use in food-animal production [106, 107]. However, even in the restricted environment in Australia, phenotypic resistance to clinically important antibiotics, including extended-spectrum cephalosporins and fluoroquinolones, has been observed in *E. coli* that belong to globally disseminated *E. coli* lineages ST744, ST100 and ST1 [108]. Globally, genomic surveillance is needed to understand the relative contribution of food-production animals to the complex web of interactions between microbiota and the mobile resistome, to provide baseline carriage rates for antimicrobial genes and VAGs, and to monitor the emergence of novel drug-resistant pathogens [84, 109].

In summary, we report, to our knowledge, the first genomic study of commensal *E. coli* isolated from commercial pigs used for food consumption and provide data to inform assessment of potential risks pig commensal *E. coli* may pose to human health. Our results show that swine are a reservoir: (i) for phylogroup A and B1 *E. coli* that carry VAGs, (ii) the *sul3* gene, (iii) class 1 integrons associated with IS*26*, and (iv) *E. coli* lineages belonging to CC10. Our study has identified several new genetic signatures that may be used in tracking mobile ARGs.

## Data bibliography

1. Zankari E, Hasman H, Cosentino S, Vestergaard M, Rasmussen S *et al.* Identification of acquired antimicrobial resistance genes. *J Antimicrob Chemother* 67, 2640–2644 (2012).
2. Siguier P, Perochon J, Lestrade L, Mahillon J, Chandler M. ISfinder: the reference centre for bacterial insertion sequences. *Nucleic Acids Res* 34, D32–D36 (2006).
3. Joensen KG, Tetzschner AM, Iguchi A, Aarestrup FM, Scheutz F. Rapid and easy *in silico* serotyping of *Escherichia coli* isolates by use of whole-genome sequencing data. *J Clin Microbiol* 53, 2410–2426 (2015).
4. Joensen KG, Scheutz F, Lund O, Hasman H, Kaas RS *et al.* Real-time whole-genome sequencing for routine typing, surveillance, and outbreak detection of verotoxigenic *Escherichia coli*. *J Clin Microbiol* 52, 1501–1510 (2014).
5. Carattoli A, Zankari E, Garcia-Fernandez A, Voldby Larsen M, Lund O *et al. In silico* detection and typing of plasmids using PlasmidFinder and plasmid multilocus sequence typing. *Antimicrob Agents Chemother* 58, 3895–3903 (2014).

## Supplementary Data

122 Supplementary File 1
